# Effective Tetradentate Compound Complexes against *Leishmania* spp. that Act on Critical Enzymatic Pathways of These Parasites

**DOI:** 10.3390/molecules24010134

**Published:** 2018-12-31

**Authors:** Kristína Urbanová, Inmaculada Ramírez-Macías, Rubén Martín-Escolano, María José Rosales, Olaf Cussó, Joan Serrano, Anna Company, Manuel Sánchez-Moreno, Miquel Costas, Xavi Ribas, Clotilde Marín

**Affiliations:** 1 Department of Parasitology, Instituto de Investigación Biosanitaria (ibs. Granada), Hospitales Universitarios de Granada/University of Granada, Severo Ochoa s/n, E-18071 Granada, Spain; kris.urbanova@gmail.com (K.U.); iramirezm@ugr.es (I.R.-M.); martinescolano@ugr.es (R.M.-E.); mjrosale@ugr.es (M.J.R.); msanchem@ugr.es (M.S.-M.); 2 QBIS-CAT Research Group, Institut de Química Computacional i Catàlisi (IQCC), and Departament de Química, Universitat de Girona. Campus de Montilivi, E-17071 Girona, Spain; olaf.cusso@udg.edu (O.C.); joan.serrano@udg.edu (J.S.); anna.company@udg.edu (A.C.)

**Keywords:** amastigote, antileishmania, antiproliferative, *Leishmania* spp., promastigote, SOD, tetradentate polyaminic compounds, ultrastructure

## Abstract

The spectrum and efficacy of available antileishmanial drugs is limited. In the present work we evaluated in vitro the antiproliferative activity of 11 compounds based on tetradentate polyamines compounds against three *Leishmania* species (*L. braziliensis*, *L. donovani* and *L. infantum*) and the possible mechanism of action. We identified six compounds (**3**, **5**, **6**, **7**, **8** and **10**) effective against all three *Leishmania* spp both on extracellular and intracellular forms. These six most active leishmanicidal compounds also prevent the infection of host cells. Nevertheless, only compound **7** is targeted against the *Leishmania* SOD. Meanwhile, on the glucose metabolism the tested compounds have a species-specific effect on *Leishmania* spp.: *L. braziliensis* was affected mainly by **10** and **8**, *L. donovani* by **7**, and *L. infantum* by **5** and **3**. Finally, the cellular ultrastructure was mainly damaged by **11** in the three *Leishmania* spp. studied. These identified antileishmania candidates constitute a good alternative treatment and will be further studied.

## 1. Introduction

Leishmaniasis are a group of complex sandfly-transmitted diseases caused by a protozoa parasite from over 20 *Leishmania* species that affect mainly the poorest population of developing countries [1]. According to World Health Organization, leishmaniasis causes 20,000–30,000 deaths and 700,000–1 million new cases annually that impose a serious health problem worldwide. They are widely spread in east Africa, Latin America, eastern Mediterranean region, south-east Asia region, and European region by importing cases mainly from Africa and America. However, the principal etiological agents, phlebotomine vectors, animal reservoirs and clinical syndromes in each continent may be different [1].

There are three predominant clinical forms. Cutaneous leishmaniasis (CL) presents itself by self-healing erythematous papules and, therefore, is non-life-threatening. Mucosal or muco-cutaneous leishmaniasis (MCL) causes deforming inflammation of the mucosa with non-self-healing ulcerations, which can disseminate through the lymphatic system. In the last, visceral or kala-azar Leishmaniasis (VL), there are no apparent ulcerations, but the symptoms are much more severe. If left untreated, it is a fatal disease characterized by irregular fever, malaise, weight loss, hepatosplenomegaly and anaemia with or without lymphadenopathy, frequently associated with secondary infections [2]. Even after a treatment, the disease reemerges or reappears as post-kala-azar dermic leishmaniasis (PKDL), causing hypopigmentation, disperse dermic or epidermic inflammation and macular, popular or nodular rash. People with PKDL are a potential source of kala-azar infection [1].

*Leishmania braziliensis* is one of the most representative species that cause MCL, and is mainly active in Brazil, Colombia, Venezuela and the Andes range [3]. On the other hand, *L. infantum* is the primary agent responsible for CL in the Mediterranean Basin (Spain, Greece and northern Africa) [4]. VL and PKDL are caused by *L. donovani*–*L. infantum* complex in the Old World and by *L. infantum* (syn. *L. chagasi*) in the New World, although PKDL is extremely rare [5]. VL has by far the greatest impact, not only because of the prevalence and lethality which placed leishmaniasis ninth in a global analysis of infectious diseases. Also, there is a growing incidence of resistance against the first-line antimony drugs and drug combinations in endemic regions [6].

The treatment depends on several factors such as type of disease, parasite species, concomitant pathologies, and geographic location [1]. Apart from the increasing drug resistance, there are other drawbacks that make the current treatment difficult, such as the administration method, a long treatment period, high toxicity and cost and limited availability in endemic regions. The spectrum and efficacy of available antileishmanial drugs is limited. The front-line drugs are pentavalent antimonial compounds (sodium stibogluconate or Pentostam and meglumine antimoniate or Glucantime), while amphotericin B, pentamidine and paromomycin (aminosidine) are used as second-line chemotherapy. Other alternative antileishmanial medicines are miltefosine, azithromycin, allopurinol, dapsone, sitamaquine, rifampicin or azole medicines: ketoconazole, fluconazole, itraconazole, none of which were designed specifically as leishmaniasis treatment. Combining the drugs in order to create a multidrug therapy may bring significant advantages and better therapeutic effects than each of the components alone [7,8]. The treatment success depends of the clinical form of leishmaniasis, the *Leishmania* spp. and the geographical region [9].

Target Product Profile (TPP), a planning tool for promising therapeutic candidates, takes into account different factors (compound efficacy, delivery mode, dosage form, time of treatment, stability, tolerability, safety, contraindications, and cost) [10]. A broad-spectrum activity, in terms of distinct species and strains, should be considered as additional specificities for VL and CL [11,12].

Therefore, given the lack of appropriate treatments and the epidemiological impact of the disease, the development of new drug candidates, safer and more effective, remains a priority. In this respect, in the last decade our group has studied many potential leishmanicidal and trypanocidal compounds, comprising natural extracts and newly synthesized compounds [13,14,15].

In our research, we try to develop molecules that are able to inhibit parasite-specific enzymes such as the iron superoxide dismutase (Fe-SOD). The Fe-SOD plays a key role in the oxidative stress defence mechanism in the trypanosomatid family, and this isoenzyme is not present in mammalian cells. The typical mammalian superoxide dismutases are linked either to manganese or to copper/zinc atoms (Mn-SOD or Cu/Zn-SOD). Mehlotra [16] has demonstrated that the parasitic protozoan survival is closely related to the ability of Fe-SOD to decompose the superoxide radical originated by the host cell. In this line, some of us have studied in depth during the last decade the redox properties of polyaminic tetradentate compounds, including their corresponding Fe and Mn-based coordination complexes, and their ability to create powerful oxidizing species upon fast reaction with hydrogen peroxide [17]. Our starting point for the present study was to test the ability of this type of compounds to cause a disruption of Fe-SOD activity, and therefore, to affect the viability of the parasite.

The major aim of the present work was to evaluate in vitro the antiproliferative activity of 11 tetradentate polyaminic compounds, fully stable in water and in cell cultures [17], against promastigote and amastigote forms of three *Leishmania* spp. (*L. braziliensis*, *L. donovani* and *L. infantum*) and their non-specific cytotoxicity against macrophages, as well as to perform infectivity assays on macrophage cells. Furthermore, we have studied the effect of the compounds on the ultrastructure of *Leishmania* promastigotes by transmission electronic microscopy (TEM), the SOD-isoenzyme specificity and the caused metabolic alterations by ^1^H-NMR analysis, specifically on the glycolytic pathway since it is the prime energy source of the parasites.

## 2. Results

### 2.1. Selection of the Compounds Under Study

We selected two Fe-based complexes **1** and **2**, four Mn-based complexes **3**–**6** and five polyamine metal-free compounds **7**–**11**. It was envisioned that the selected metal based compounds could generate oxidizing species that may elude the protecting role of the Fe-SOD enzyme of the parasite. On the other hand, the metal-free compounds were selected to understand the effect of the metal in some cases (compounds **7**, **9**, **11**) whereas **8** and **10** were tested for their known ability to strongly chelate transition metals, aiming at disrupting the parasite metabolism by sequestering transition metals essential for their viability, such as iron.

### 2.2. In Vitro Antileishmanial Activity

**Figure 1:**
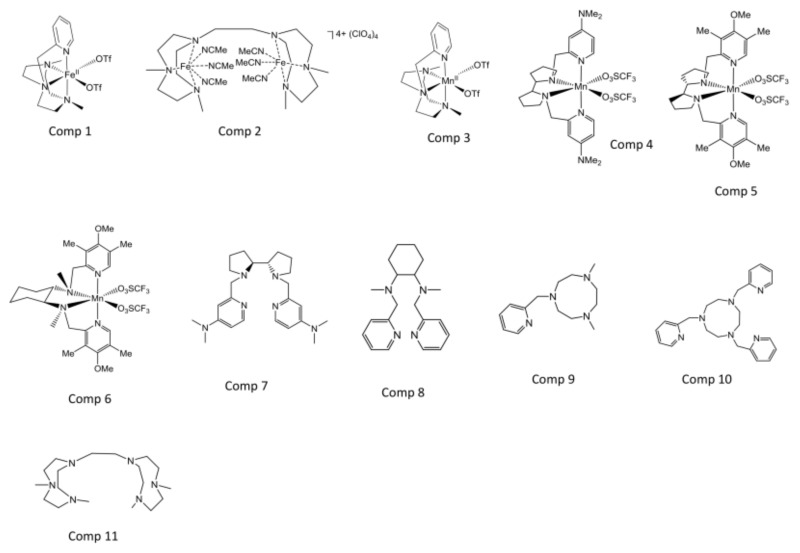
Chemical structure of the tested polyaminic compound complexes.

We have tested the in vitro inhibitory properties of 11 polyaminic compound complexes (Figure 1) against the extra- and intracellular forms of three *Leishmania* spp., *L*. *braziliensis*, *L*. *donovani* and *L*. *infantum*. The IC_50_ values for promastigote and amastigote forms were calculated by non-linear regression analysis from Kc values at concentrations ranging between 1 and 100 µM and are shown in Table 1A, which also includes the IC_50_ of Glucantime (Glu), a frequently used reference drug. Also, we have determined the toxicity values against J774.2 murine macrophages. The selectivity index values (SI, IC_50_ macrophage cell toxicity/IC_50_ of promastigote or amastigote forms of the parasite) and the times by which the tested compounds exceed the SI value of Glu are shown in Table 1B. Taking into consideration the fact that amastigote forms are the ones present in mammalian hosts, the IC_50_ and SI achieved against this parasitic form were determining for us to carry on with subsequent assays.

We found that six compounds (**3**, **5**, **6**, **7**, **8** and **10**) were effective against all three *Leishmania* spp. (Table 1). However, **3** remained slightly below the cut-off value (drug SI 20 or more times higher than that of the reference drug as hit activity criteria proposed by [18]) for *L. donovani*. Five compounds (**3**, **5**, **6**, **7**, and **10**) presented 2 to 30-fold lower IC_50_ values against amastigotes than Glu in all three *Leishmania* spp. Still, we could observe some species-specificity in the in vitro effectiveness of the compounds. Compounds **6**, **7** and **8** were from 8 to 11-fold more effective against *L. donovani* amastigotes than against *L. braziliensis* and *L. infantum* amastigotes. On the other hand, **3** and **5** manifested an efficacy 2 and 4 times higher in *L. infantum* amastigotes compared to the other *Leishmania* spp. The last compound of this subseries, **10**, was equally effective in all three target species. All subsequent tests were carried out for only the most leishmanicidal compounds (**3**, **5**, **6**, **7**, **8** and **10** for *L. braziliensis* and *L. infantum*, and **5**, **6**, **7**, **8** and **10** for *L. donovani*).

**Figure 2:**
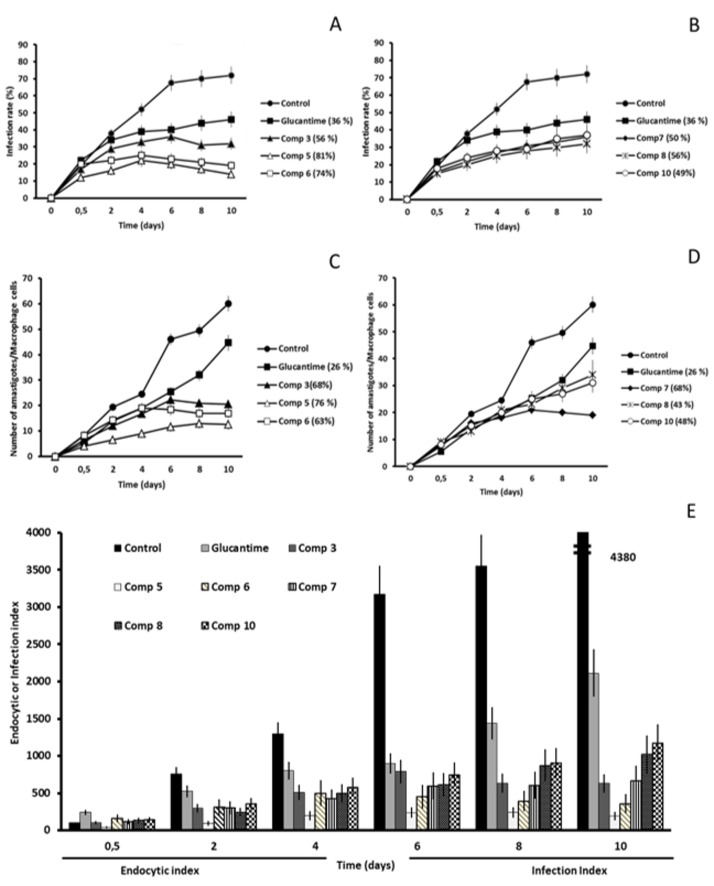
*Leishmania braziliensis* infectivity assay. (**A**,**B**) Infection rate percentage and (**C**,**D**) amastigote count per macrophage cell. In brackets: percentage of decrease in infection rate/number of amastigotes per macrophage cell in comparison to the control measured on the last day of the experiment (peak of infection). (**E**) Effects of the compounds on the endocytic and infection indexes. Each drug at IC_25_ concentration was tested in triplicate. Values are the means of four separate determinations ± standard deviation.

**Figure 3:**
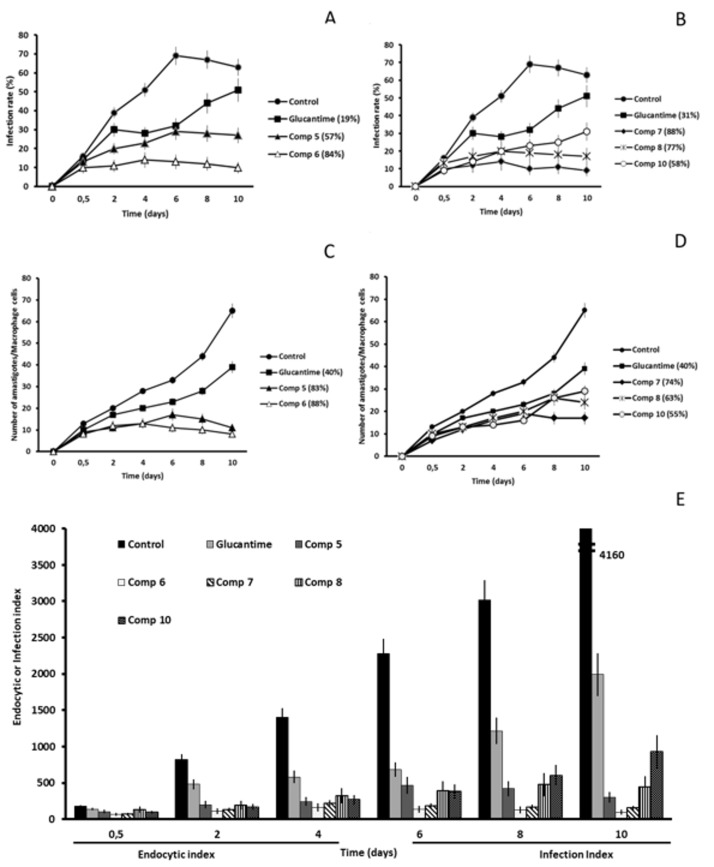
*Leishmania donovani* infectivity assay. (**A**,**B**) Infection rate percentage and (**C**,**D**) amastigote count per macrophage cell. In brackets: percentage of decrease in infection rate/number of amastigotes per macrophage cell in comparison to the control measured on the last day of the experiment (peak of infection). (**E**) Effects of the compounds on the endocytic and infection indexes. Each drug at IC_25_ concentration was tested in triplicate. Values are the means of four separate determinations ± standard deviation.

**Figure 4:**
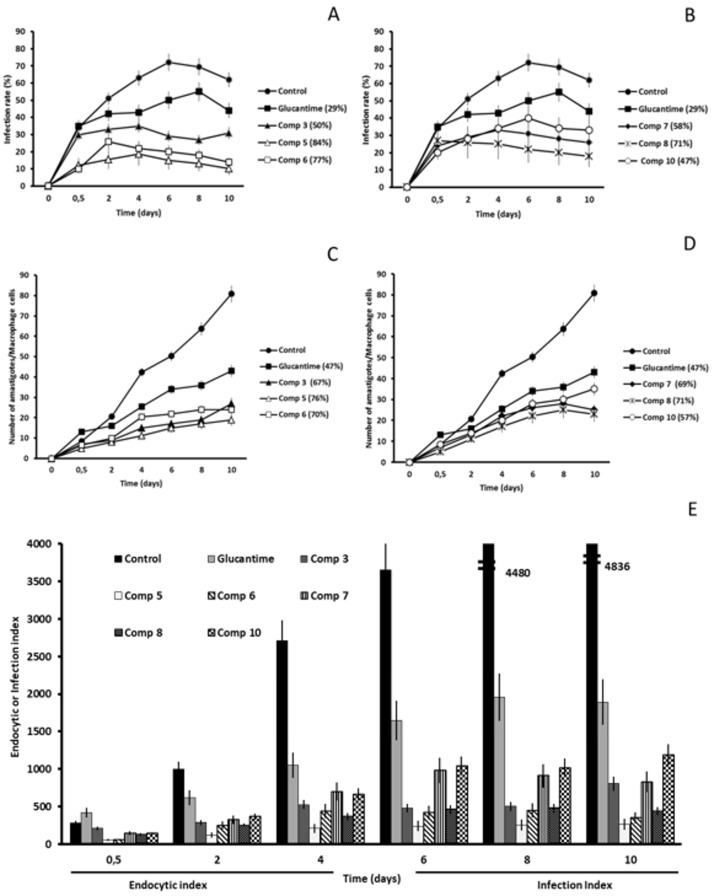
*Leishmania infantum* infectivity assay. (**A**,**B**) Infection rate percentage and (**C**,**D**) amastigote count per macrophage cell. In brackets: percentage of decrease in infection rate/number of amastigotes per macrophage cell in comparison to the control measured on the last day of the experiment (peak of infection). (**E**) Effects of the compounds on the endocytic and infection indexes. Each drug at IC_25_ concentration was tested in triplicate. Values are the means of four separate determinations ± standard deviation.

After the initial in vitro studies we were interested in finding out if the six most leishmanicidal compounds could also prevent the infection of host cells. For this reason, we conducted an assay in which the J774.2 macrophages were exposed to the metacyclic promastigotes and the compound at IC_25_ concentration at the same time. The propagation of the parasitic infection was determined by measuring the infection rate and the average number of amastigotes per macrophage cell present during the 10-day treatment period. In addition, the endocytic (short-time of infection) and infection (long-time of infection) indexes were calculated. The control samples did not include any inhibitory compounds, so, the last day the infection rates in *L. braziliensis*, *L. donovani* and *L. infatum* reached 72, 63 and 62%, respectively (Figure 2A, Figure 3A and Figure 4A).

In *L. braziliensis*, the two compounds that caused the highest infectivity inhibition were **5** and **6** (inhibition of 81 and 74%) that caused a decrease in amastigote count of 76 and 63%, respectively. As is shown in Figure 2, all the compounds were much more effective than the reference drug, Glu (infectivity reduction of 36%). These two compounds were the most effective against *L. infantum*, as well, originating an infectivity inhibition of 84 and 77%, and an average amastigote count reduction of 76 and 70% (Figure 4).

The compound that manifested the highest inhibitory capacities was **7** against *L. donovani* (Figure 3). It confirmed its inhibitory capacities against *L. braziliensis* and *L. infantum*, but the effect was not so powerful, confirming the species-specificity we suspected. The infectivity reduction reached 88% and caused an amastigote per macrophage cell count reduction of 74%. In general, the onset of all compounds against the subgenus Viannia (*L. braziliensis*) was slower than against Leishmania (*L. donovani* and *L. infantum*). The decline in the infection rate of *L. braziliensis* started to appear between days 4 and 6 (Figure 2A,B).

However, the same reduction of subgenus Leishmania (Figure 3A,B and Figure 4A,B) appeared between day 2 and 4 for most compounds, with some noticeable exceptions (day 2 for 5, 6 and 7 against *L. donovani*, or 0.5 days for 8 against *L. infantum*).

Evidently, in view of the previous results, the leishmanicidal activity of the compounds was also visible in endocytic and infective indexes (Figure 2E, Figure 3E and Figure 4E) from the beginning of the treatment, and maintained until the day 10 after treatment. Moreover, all compounds tested showed endocytic and infective indexes lower than those obtained for Glu for the three *Leishmania* spp. Because the inhibitory effect of all compounds was evident only 2 days after treatment, we can suggest that these leishmanicidal compounds are fast killing drugs. It is worth mentioning that all compounds tested against three *Leishmania* spp. in the present research study exhibited an infection rate capacity up to 3 times higher than Glu.

### 2.3. Studies on the Mechanism of Action

We performed several experiments to elucidate a possible mechanism of action for the six most active tetradentate compound complexes, according to the SI.

#### 2.3.1. SOD Inhibition

The previous extraordinary results prompted us to evaluate the inhibitory effect of **3**, **5**–**8** and **10** on the SOD activity. We were looking for a compound that would selectively inhibit parasitic SODs and not their human equivalent. We used promastigotes of *Leishmania* spp. to obtain Fe-SOD by cellular disruption and precipitation and assayed the compounds at concentrations ranging from 100 µM to 0.1 µM in each case.

**Figure 5:**
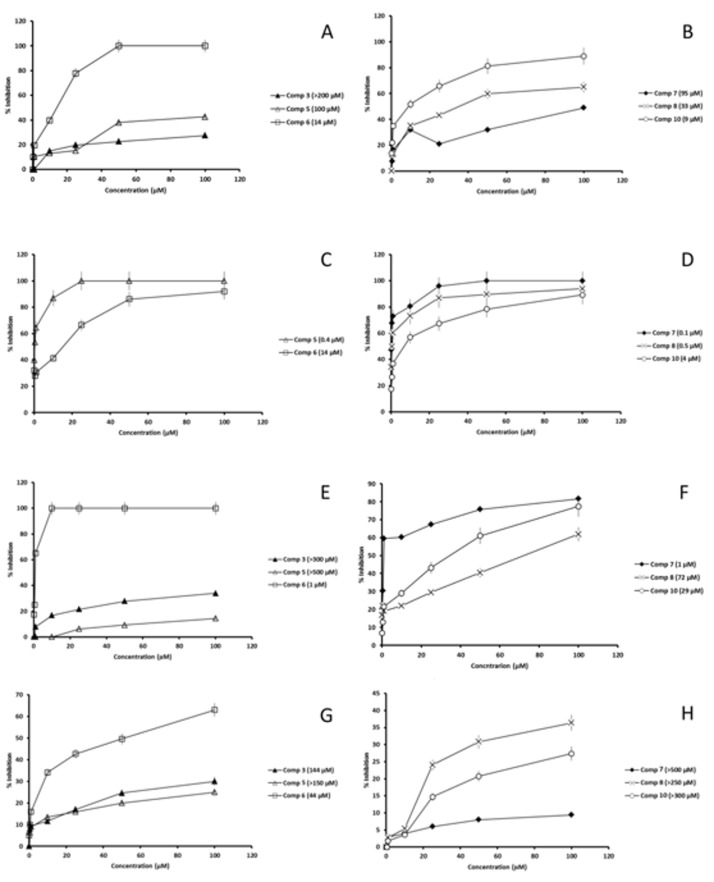
SOD inhibition assay. Representation of the inhibition of **3**, **5**–**8** and **10** against: (**A**,**B**) *L. braziliensis* Fe-SOD (**C**,**D**) *L. donovani* Fe-SOD (**E**,**F**) *L. infantum* Fe-SOD and (**G**,**H**) human CuZn-SOD. Each drug concentration was tested in triplicate. Values are the means of four separate determinations ± standard deviation. In brackets: IC_50_ value, calculated by non-linear regression analysis.

The results are shown in Figure 5. The corresponding IC_50_ values were calculated and are included in the same figure. Not all compounds were equally effective against the three *Leishmania* spp. As can be concluded from the figure, 3 is not an SOD inhibitor, since its IC_50_ values exceeded 100 µM in both parasitic Fe-SOD and human CuZn-SOD, even causing a greater inhibition in the human isoform. 5 caused notable inhibition in *L. donovani* Fe-SOD (IC50 = 0.4 µM) but not in other *Leishmania* spp. 6 is undoubtedly a potent SOD inhibitor and it showed preference for the parasitic isoforms like we were looking for. It produced an inhibition from 3 to 44-fold higher than the one of human SOD. The last three compounds (7, 8 and 10) did not inhibit the CuZn-SOD in the same extent as the Fe-SOD of *Leishmania* spp. As it is shown in Figure 5B,D,F, these compounds are very powerful Fe-SOD inhibitors: from 3 (8 against *L. infantum* Fe-SOD) to 5000 times (**7** against *L. donovani* Fe-SOD) more effective than against CuZn-SOD.

The most effective compound against *L. braziliensis* Fe-SOD was **10** and against *L. infantum* Fe-SOD were **6** and **7**. **7** was also the overall most potent Fe-SOD inhibitor against all *Leishmania* spp., but particularly against *L. donovani*. Furthermore, it is the least toxic compound for the human isoform.

#### 2.3.2. ^1^H-NMR

**Figure 6:**
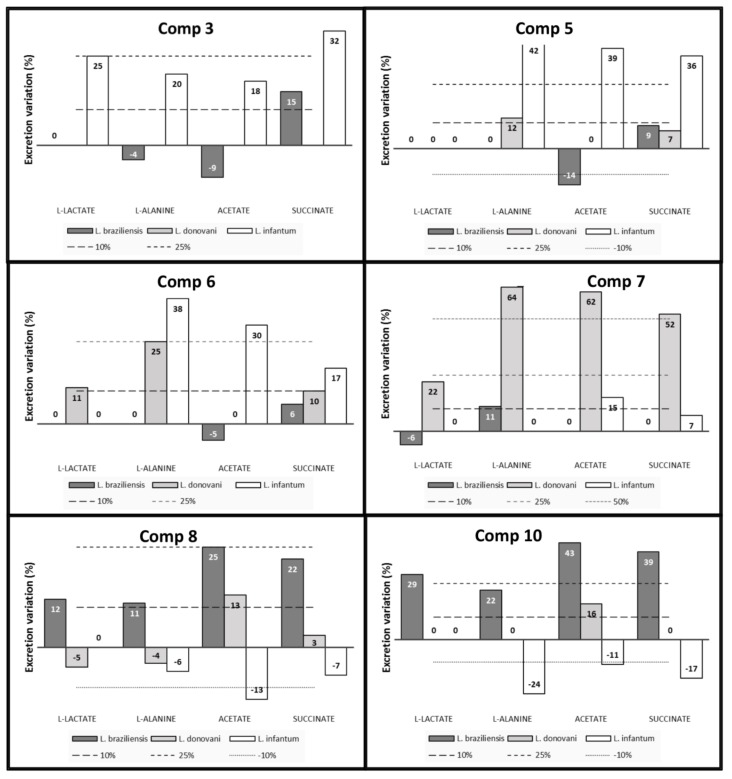
Variation in metabolic excretion in *L. braziliensis*, *L. donovani* and *L. infantum* exposed to compounds at IC_25_ concentrations in comparison to untreated incubated 72 h, and determined by ^1^H-NMR.

Trypanosomatids catabolizes glucose at a high rate, excreting into the medium partly oxidized end products such as acetate, succinate, L-alanine or L-lactate [19,20]. Therefore, we cultivated the promastigotes of *L. braziliensis*, *L. donovani* and *L. infantum* in glucose-rich medium (MTL) for 96 h with and without the six most effective compounds (**3**, **5**, **6**, **7**, **8** and **10**) at IC_25_ concentrations to see if the compounds altered the usual excretion pattern, and consequently, altered the glucose metabolism. ^1^H-NMR spectroscopy was used to identify and quantify the fermented metabolites in cell-free media after parasite culture and to compare with those metabolites found for untreated parasites. Figure 6 shows the variation in metabolic excretion of the four most important excreted metabolites (d-lactate, l-alanine, acetate and succinate). We considered a variation superior to 10% as significant.

Compound **3** was not assayed against *L. donovani* for reasons described above. This compound raised the levels of all excreted metabolites in *L. infantum* (from 18% for acetate to 32% for succinate), but only one in *L. braziliensis* (succinate). This is in agreement with the fact that succinate is the major excreted metabolite in *Leishmania* spp. [13]. Compound **5** did not affect lactate levels in any *Leishmania* spp. Even we could say that the only affected species is, again, *L. infantum* (increase in L-alanine by 42%, in acetate by 39% and in succinate by 36%). Once more, the **6** affected mostly the metabolism of *L. infantum*, causing an increase in L-alanine, acetate and succinate, but in a lesser extent this time.

Both compounds, **5** and **6**, caused an increment in L-alanine (12 and 25%, respectively) in *L. donovani*. As we said above, the star compound against *L. donovani* assayed in this study was **7**. Once again, the most affected metabolite was L-alanine (increase in 64%), but this time acetate and succinate were also extremely elevated (superior to 50%). Other *Leishmania* spp. were not affected in the same amount.

The two last compounds, **8** and **10**, were the most effective against *L. braziliensis* promastigotes, altering all detected metabolites from 22 to 43%. Both elevated the acetate excreted by *L. donovani* slightly and decreased the same metabolite in *L. infantum*. **10** also decreased the levels of alanine and succinate in *L. infantum*.

#### 2.3.3. Microscopy

The ultra-thin sections of promastigotes of *L. braziliensis*, *L. donovani* and *L. infantum* were examined at transmission electron microscopy. The promastiogotes were treated with the most effective compounds in each *Leishmania* spp.

**Figure 7:**
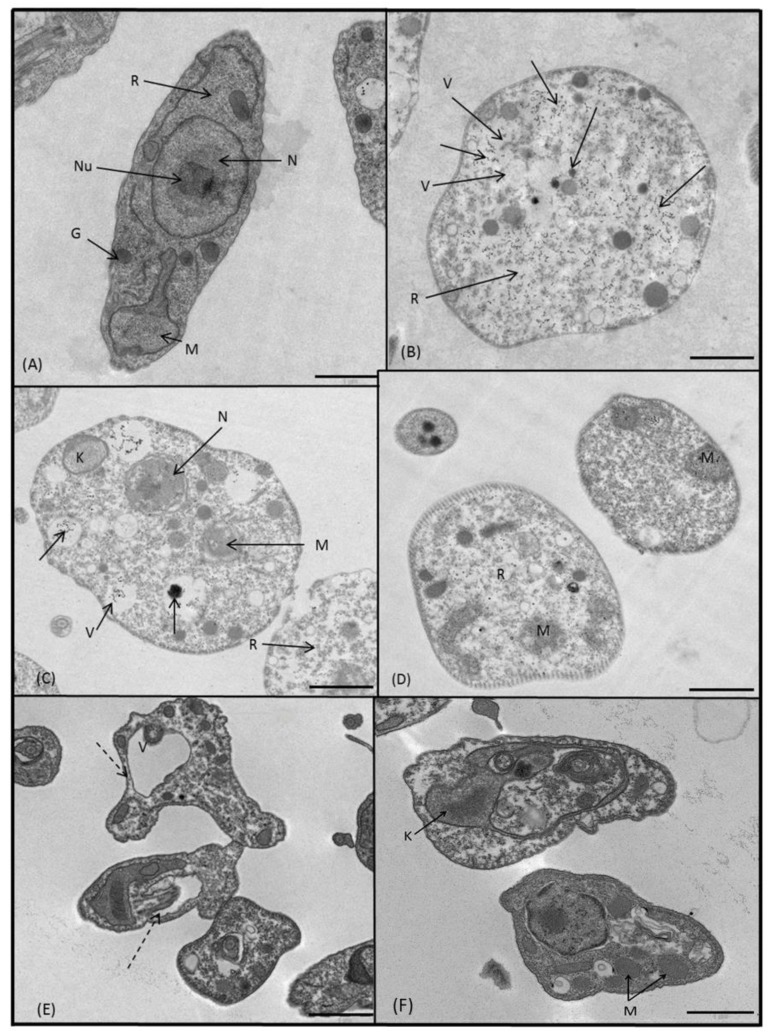
Ultrastructural images of *L. braziliensis* promastigotes treated for 72 h at IC_25_ concentrations with: (**A**) untreated control, (**B**) **3**, (**C**) **7**, (**D**) **8**, (**E**,**F**) **10**. (N) nucleus, (Nu) nucleolus, (R) ribosomes, (K) kinetoplast, (M) mitochondrion, (G) glycosome, (V) vacuole, (arrow) electron-dense precipitates of unassimilated compounds, (dashed arrow) irregular morphologies. In all cases scale bar corresponds to 1 μm.

The promastigotes of *L. braziliensis* were treated with the compounds **3**, **5**–**8**, and **10** (Figure 7B–F), and we observed quite a lot of alterations in comparison to the untreated control (Figure 7A), with exception of **5** and **6** which did not cause apparent damage. The promastigote treated with **3** (Figure 7B) is completely swollen, round and full of empty or lipid vacuoles. In addition, there are a lot of electron-dense granules and a notable absence of ribosomes. In Figure 7C, we show a similar effect on the parasite caused by **7**. The promastigote seems to be damaged, swollen, and full of vacuoles. We observed swollen mitochondria, disintegrated kinetoplasts and compound precipitates in the cytoplasm but not in the exterior in all examined plates. These electron-dense precipitates were present in the promastigotes treated with **8**, also (Figure 7D). The promastigotes were round and lacked in ribosomes, apart from unrecognizable or swollen mitochondria. The bizarre effect of the last compound, **10**, on *L. braziliensis* is shown in Figure 7E–F. The parasites seemed to be shrunken or dehydrated, had irregular morphologies, global electron density and a wavy plasmatic membrane. There were countless vacuoles (Figure 7F) in the cytoplasm and the kinetoplasts and mitochondria were swollen, deformed and unrecognizable, and apparently had no mitochondrial cristae.

**Figure 8:**
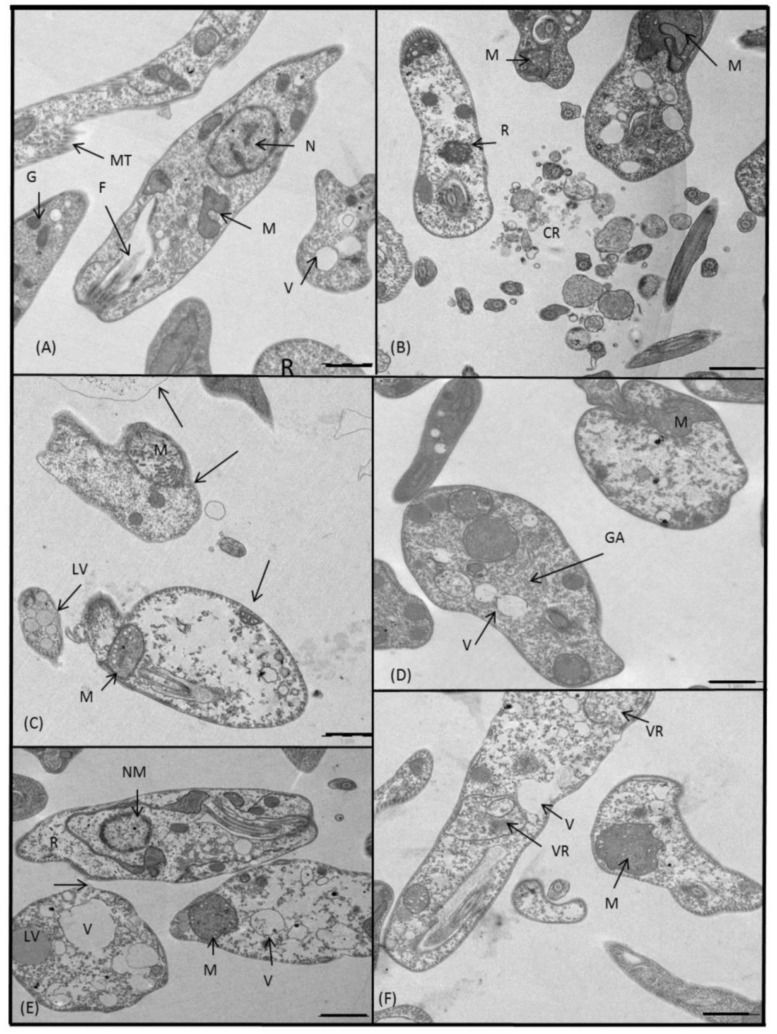
Ultrastructural images of L. donovani promastigotes treated for 72 h at IC_25_ concentrations with: (**A**) untreated control, (**B**) **6**, (**C**) **7**, (**D**) **8**, (**E**) **10**, (**F**) **5**. (N) nucleus, (NM) nuclear membrane, (M) mitochondrion, (F) flagellum, (R) ribosomes, (MT) microtubule, (GA) Golgi apparatus, (G) glycosome, (CR) cellular rest, (V) vacuole, (LV) lipid vacuole, (VR) vacuole full of rests, (arrow) dead promastigote. In all cases scale bar corresponds to 1 μm.

Equally, we studied the ultrastructural alterations caused by **5**–**8** and **10** on in vitro cultured L. donovani promastigotes and compared them to the control (Figure 8A). All tested compounds confirmed a similar toxic effect as was described above. The promastigotes after the treatment with **6** (Figure 8B) were still alive, but very close to death, showing either altered organelles and no ribosomes, or many empty vacuoles. In each case, there was a significant damage to the structure of the mitochondria, and on top of that, they were full of small vesicles and possessed no cristae. The effect of **7** was very similar in all three *Leishmania* spp.: a cytoplasmic emptiness, none or a few ribosomes, swollen and disintegrated mitochondria and abundant lipid and non-lipid vacuoles (Figure 8C). Promastigotes treated with **8** (Figure 8D), in addition to the damage akin to that described for **7**, contained an expanded and disperse Golgi apparatus. **10** (Figure 8E) exerted the most lethal end result in L. donovani promastigotes. Dead parasites underwent a nuclear and cytoplasmic lysis, and they were full of lipid vacuoles. Similarly, **5** (Figure 8F) was equally effective as the latter one, causing ribosomal destruction, an intense vacuolization and augmentation of mitochondria size by 4 times which stood out among other alterations.

**Figure 9:**
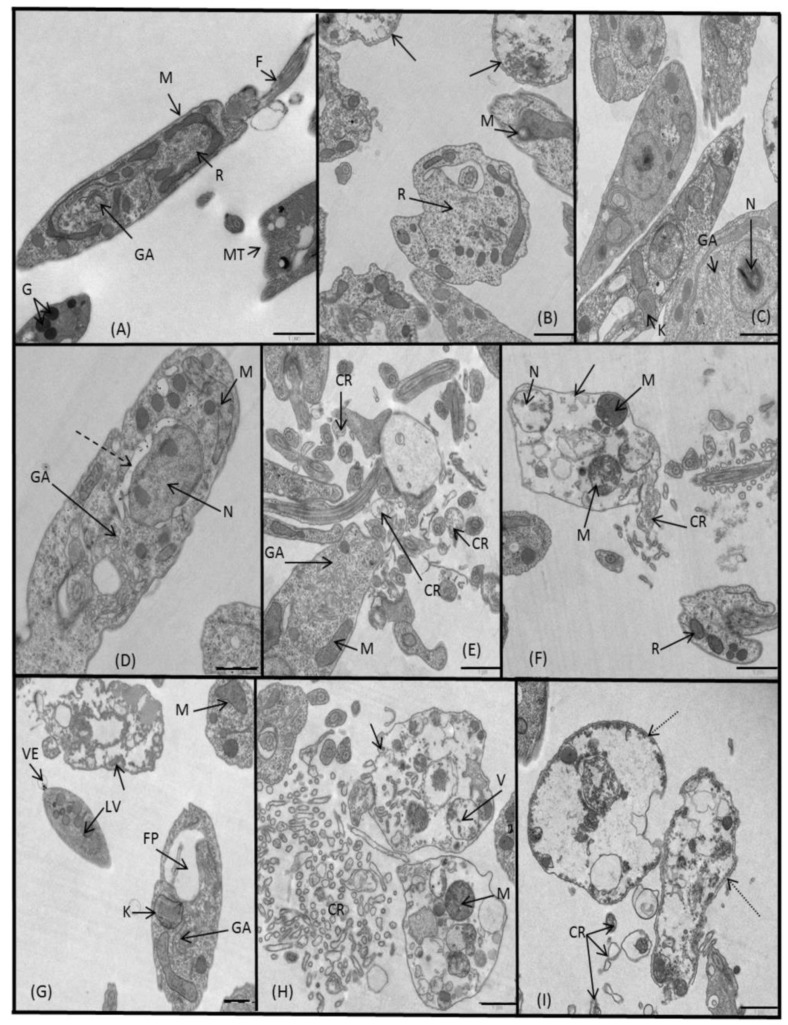
Ultrastructural images of *L. infantum* promastigotes treated for 72 h at IC_25_ concentrations with: (**A**) untreated control, (**B**,**C**) 3, (**D**,**E**) 5, (**F**) 7, (**G**,**H**) 8, (**I**) **10**. (N) nucleus, (M) mitochondrion, (F) flagellum, (R) ribosomes, (MT) microtubule, (GA) Golgi apparatus, (G) glycosome, (K) kinetoplast, (CR) cellular rest, (V) vacuole, (LV) lipid vacuole, (VE) vesicle, (FP) flagellar pocket, (arrow) dead promastigote, (dashed arrow) nuclear membrane separation, (dotted arrow) lysed promastigote. In all cases scale bar corresponds to 1 μm.

Lastly, we studied the alterations on *L. infantum* promastigotes by TEM. Generally, every essayed compound (**3**, **5**, **7**, **8** and **10**) was effective and caused noteworthy structural changes. Compound **3** induced agglutination in the parasites, some of which were dead and other were fading, as it is witnessed by a deficiency in ribosomes and by a presence of distended mitochondria filled with vacuoles and a swollen kinetoplast (Figure 9B,C). Additionally, we noticed an intense vacuolization, an appearance of concentric myelinic figures which originated most probably from the degenerated mitochondria, an enlarged Golgi apparatus and an abnormal electron-dense structure in the nuclei. A similar mitochondrial swelling was observed after treatment with **5** (Figure 9D,E), besides hypertrophic Golgi apparatus and nuclear membrane separation. The most destructive compounds were **7**, **8** and **10**, causing an almost identical outcome. We noticed cytoplasmic membrane ruptures, abundance of vacuoles, a general lack in ribosomes, swollen mitochondria, disintegrated nuclei and no cytoplasmic content. The parasites treated with **8** and **10** (Figure 9G,H,I, respectively) showed swelling in mitochondria, Golgi apparatus and flagellar pocket, copious lipid vacuoles and in some parasites, a vesicle attached to the cytoplasmic membrane.

## 3. Discussion

The goal of the present study was to test the ability of Fe and Mn-based bearing polyaminic ligands, known for their rich redox properties [21,22,23,24], and of the polyamine ligands themselves strong metal chelators, to affect the viability of the parasite.

The best compounds were **3**, **5**, **6**, **7**, **8** and **10** which exhibit a remarkable antileishmania effect and absence of toxicity. Moreover, all assayed compounds were much more effective than the reference drug (Glu) to prevent the infection of host cells, acting as fast killing drugs. We detected a subgenera-specificity in regard to the infection. All compounds show a sooner onset against subgenus Leishmania (*L. donovani* and *L. infantum*) than against subgenus Viannia (*L. braziliensis*). We suspect that the difference in protein composition in the membrane [25] of the subgenera may be the cause, but we will have to investigate this aspect further in the future. A species-specificity of the compounds is apparent because **5** and **6** impeded the cell infection by *L. braziliensis* and *L. infantum* the most, while **7** was most successful against *L. donovani*.

Regarding the mechanism of action, the most remarkable difference for **3** was the altered amount of succinate for *L. infantum*. It is well-known that this increase can indicates a redox stress [26], and not a direct effect on the glucose metabolism: the main role of succinate is to maintain the redox balance in the glycosoma via NADH reoxidation, and it is possible that this pathway is increased to maintain it, with the consequent increase and excretion in succinate as final product [10,23,24,27]. **5** produces a similar effect for *L. infantum*, and the excretion of l-alanine and acetate can also be increased as a result of the enhancement of the succinate pathway [23].

Compound **6** affected mostly the metabolism of *L. infantum* and *L. donovani*, causing an increase in L-alanine, acetate and succinate, but in a lesser extent this time. Since this compound is a powerful inhibitor of the Fe-SOD of *L. infantum*, the metabolic alteration can be also a consequence of a mitochondrial dysfunction associated to this enzymatic inhibition.

As we said above, the star compound against *L. donovani* assayed in this study was **7**. Once again, the most affected metabolites were L-alanine, acetate and succinate. These results can indicates, as before, a redox stress. In addition, this compound **7** is a potent Fe-SOD inhibitor that can cause a mitochondrial dysfunction and be the final cause of the alteration in the metabolic excretion. The singular behavior of this compound can be tentatively traced to a combination of its strong chelate nature and its powerful electron donating character, conferred by the dialkylamine moiety. This molecule has the potential ability to sequester the iron metal center of SOD, disrupting its activity, and can also generate iron complexes highly active in red-ox processes, further promoting the oxidative estress.

The two last compounds, **8** and **10**, were the most effective against *L. braziliensis* promastigotes, altering all detected metabolites. The reasons can be similar to what happens in *L. infantum* for **3** and **5**. This effect can be traced to the strong chelating nature of these polyamines, which may sequester transition metal ions, generating red-ox active species that disturb the red-ox balance.

Finally, visible ultrastructural alterations showed a cytoplasmic emptiness, none or a few ribosomes, swollen and disintegrated mitochondria and abundant lipid and non-lipid vacuoles, an appearance of concentric myelinic figures which originated most probably from degenerated mitochondria, an enlarged Golgi apparatus and an abnormal electron-dense structure and disintegrated nuclei. These cytoplasmic effects could be the result of an ineffective metabolization of the compound by the promastigotes, causing its precipitation in the cytoplasm, mitochondrion damage and an excessive activation of the Golgi machinery to produce new vesicles which could isolate the polyamines. The notable absence of ribosomes also indicates poor survival possibilities of the promastigotes.

It should be noted that the alterations observed in the mitochondria may be both consequence of the damage produced by the inhibition of Fe-SOD for compounds **6** and **7**, as consequence of the powerful oxidizing of these compounds [17] that cause lipid peroxidation and macromolecule destruction. Then, both may be the cause of the alterations observed in the glucose metabolism.

## 4. Materials and Methods

### 4.1. Compounds Under Study

Most of our compounds (**1**, **3**, **4**, **5**, **6**, **7**, **8**, **9** and **10**) have been previously synthesized for organic synthesis purposes [21,22,28,29,30,31,32]. Compound **11** was purchased from Catexcel (Derby, UK). On the other hand, **2** has been designed as a structural variant of **11** and synthesized for the first time in this work. The respective structures are shown in Figure 1.

#### Synthesis of Compound **2**

Compound **2** was synthesized as follows: TACN2 (labelled as **11**; 47.1 mg, 0.14 mmol) was dissolved in acetonitrile (1 mL). A solution of FeCl_2_ (36.2 mg, 0.28 mmol) in acetonitrile (2 mL) was prepared under inert atmosphere. The solution of FeCl_2_ was added dropwise at room temperature to the solution of TACN2, the mixture turned pale yellow and was stirred at room temperature for 1 h. Then, AgClO_4_ (114.7 mg, 0.55 mmol) was added and the formation of a white precipitate was observed. The mixture was stirred over 30 min. Afterwards a white powder and a purple solution were clearly distinguished. The purple solution was filtered through syringe filters (25 mm Ø) and the white precipitate was washed with CH_3_CN until the filtrates became colorless. 121 mg of purple crystals (0.11 mmol, 80%) were obtained after diffusion with diethyl ether. HRMS (CH_3_CN): calcd. for [(C_18_N_6_H_40_)Fe_2_(ClO_4_)_3_]^+^ 749.0466, found 749.0461 (Supplementary data 1). ^1^H-NMR (300 MHz, CD_3_CN): 107.9, 101.1, 63.5, 37.2, 35.0, 32.1, 4.5 ppm (Supplementary data 2). Anal Calcd. for [(C18H40N6)Fe(CH3CN)3](ClO4)4: C, 29.62%; N, 12.95%; H, 5.07%. Found: C, 28.9%; N, 12.95%; H, 5.08%. X-Ray structure: **2** has the formula: [C_30_ H_58_ Fe_2_ N_12_](ClO_4_)_4_·(CH_3_CN), with the following Unit Cell Parameters: a 11.570(5) b 14.555(6) c 17.298(7), space Group P-1. Crystal data has been deposited with the Cambridge Crystallographic Database with the CCDC reference number 1047857 (Supplementary Material 3).

### 4.2. Parasite Strain and Culture

Promastigote forms of three different *Leishmania* spp., *L. donovani* (MHOM/PE/84/LC26), *L. infantum* (MCAN/ES/2001/UCM-10) and *L. braziliensis* (MHOM/BR/1975/M2904), were cultured in vitro in Medium Trypanosomes Liquid (MTL) supplemented with 10% inactivated foetal calf serum (FCS) in an air atmosphere at 28 °C, in Roux flasks (Corning, Manassas, VA, USA) with a surface area of 75 cm^2^, according to [33].

### 4.3. In Vitro Screening Assays

The compounds and the reference drug Glu were dissolved in DMSO (Panreac, Barcelona, Spain) and tested from 100 µM to 1 µM with 0.1% (*v*/*v*) DMSO final concentration in plates. Therefore, controls were prepared including 0.1% (*v*/*v*) of this component, as previously described [33].

#### 4.3.1. Cell Culture and Cytotoxicity Tests

The macrophage line J774.2 [European collection of cell cultures (ECACC) number 91051511] was derived in 1968 from a tumour in a female BALB/c mouse. Macrophages were grown in humidified 95% air, 5% CO_2_ atmosphere at 37 °C in Minimal Essential Medium (MEM) with 2 mM glutamine and 20% (*v*/*v*) inactivated FCS. The cytotoxicity test on macrophages was performed by Flow cytometry analysis according to a method previously described [34]. Briefly, cells were seeded at a density of 5 × 10^4^ cells/well in 24-well microplates (Nunc, Naperville, IL, USA). After 48 h incubation, the medium was removed, and fresh medium was added together with each test compound at concentrations from 100 µM to 1 µM and cultured in 500 μL/well volumes for 72 h. Thereafter, cells were recovered by centrifugation, and cell viability was determined by Flow cytometry (FACS Vantage flow cytometer, Becton Dickinson, San Jose, CA, USA) after incubating the cells with propidium iodide (100 mg/mL) and fluorescein diacetate (100 ng/mL). The percentage of viability was calculated in comparison with the control cultures. The toxicity is expressed as the IC_50_ value and was calculated using linear regression analysis from the Kc values of the concentrations employed (100-1 µM) [35]. Each drug concentration was tested in triplicate in four separate determinations.

#### 4.3.2. Promastigote Tests: Extracellular Forms

Promastigote forms were collected in the exponential growth phase and seeded at 1 × 10^5^ parasites/mL in 24-well microplates (Nunc, Naperville, IL, USA), after adding each compound at concentrations from 100 µM to 1 µM and cultured in 500 μL/well volumes at 28 °C. After 72 h incubation, the leishmanicidal activity was assessed using a Neubauer haemocytometric chamber in comparison with the control cultures. The leishmanicidal effect is expressed as the IC_50_ value and was calculated using linear regression analysis from the Kc values of the concentrations employed (100-1 µM) [35]. Each drug concentration was tested in triplicate in four separate determinations.

#### 4.3.3. Amastigote Tests: Intracellular Forms

J774.2 macrophages were grown and seeded at a density of 1 × 10^4^ cells/well in 24-well microplates (Nunc) with rounded coverslips on the bottom and cultured for 48 h in humidified 95% air, 5% CO_2_ atmosphere at 37 °C in Minimal Essential Medium (MEM) with 2 mM glutamine and 20% (*v*/*v*) inactivated FCS. Adherent macrophages were infected with metacyclic promastigotes in the stationary growth phase of *L. donovani, L. infantum* and *L. braziliensis* at a multiplicity of infection (MOI) ratio of 1:10 and maintained for 24 h. Non-phagocytosed parasites were removed by washing, and after addition of the testing compounds at concentrations from 100 µM to 1 µM, cultured in 500 μL/well volumes. Control cultures were also included. After 72 h incubation, leishmanicidal activity was assessed based on the number of amastigotes in treated and untreated cultures in methanol-fixed and Giemsa-stained preparations by analyzing 500 host cells distributed in randomly chosen microscopic fields. The leishmanicidal effect was calculated as described to determine the leishmanicidal activity in the promastigote forms [35]. Each drug concentration was tested in triplicate in four separate determinations.

### 4.4. Infectivity Assay

Adherent macrophages were grown, seeded and infected with metacyclic promastigotes as mentioned above. The compounds at IC_25_ concentrations (calculated using linear regression analysis from the Kc values of the concentrations employed) were added immediately after infection and incubation was performed for 12 h at 37 °C in humidified 95% air, 5% CO2 atmosphere. in humidified 95% air, 5% CO_2_ atmosphere at 37 °C Non-phagocytosed parasites and drugs were removed by washing, and then the infected cultures were grown for 10 days, adding fresh culture medium every 48 h, according to [35]. Amastigotes per macrophage count was determined in methanol-fixed and Giemsa-stained preparations as mentioned above every 48 h. The infection rate (defined as the percentage of infected cells), the average number of amastigotes per macrophage, and the endocytic and infection indexes (calculated by multiplying the infection rate by the average number of amastigotes per macrophage [36]) were determined. Each drug concentration was tested in triplicate in four separate determinations.

### 4.5. Ultrastructural Alterations

Parasites were cultured at a density of 5 × 10^5^ cells/mL with medium containing the tested compounds at IC_25_ concentrations. After 72 h at 28 °C, the cultures were processed using a technique previously described [33] and analysed by TEM (LIBRA 120 PLUS, Zeiss, Barcelona, Spain).

### 4.6. Metabolite Excretion

Cultures of *L. donovani, L. infantum* and *L. braziliensis* promastigotes were seeded in culture flaks at 5 × 10^5^ cells/mL, and after the addition of the compounds at IC_25_ concentrations at 28 °C. Control cultures were also included. After 72 h of incubation, parasites were centrifuged at 400 × g for 10 min, and supernatants were collected in order to determine the excreted metabolites by ^1^H-NMR. Chemical shifts were expressed in parts per million (ppm) using sodium 2,2-dimethyl-2-silapentane-5-sulphonate as the reference signal. The One-dimensional ^1^H-NMR spectra were acquired with a VARIAN DIRECT DRIVE 400 MHz Bruker spectrometer (Rheinstetten, Germany) with an AutoX probe using D_2_O as solvent. The chemical shifts used to identify the respective metabolites were consistent with those described previously by some of the authors [37]. The spectral region of 1.0–5.5 ppm was bucketed into a frequency window of 0.1 ppm. The peak (2.6 ppm) corresponding to DMSO was removed before binning, and the regions corresponding to water (4.5–5.5 ppm) and glucose (3.4–3.8 ppm) were excluded during binning to avoid artefacts due to pre-saturation. The aromatic region was excluded because the signal to noise ratio in this region was poorer in comparison to the aliphatic region. The resulting integrals were normalised to the working region (1.0–3.4) ppm of the spectrum to correct for inter-sample differences in dilution. The binning and normalisations were achieved using Mestrenova 9.0 software (Santiago de Compostela, Spain). The matrix obtained in Mestrenova was imported into Microsoft Excel (Redmond, WA, USA) for further data analyses.

### 4.7. Fe-SOD Enzymatic Inhibition

Parasite cells were collected in the exponential growth phase by centrifugation (400g for 10 min). The pellet obtained after centrifugation was lysated by sonication, processed according to [38] and obtained the homogenate fraction which contained the parasite Fe-SOD. The protein content was quantified using the Sigma Bradford test, which uses BSA as a standard (no trace ability was certified for the BSA standard) [39]. Superoxide dismutase activities were determined using a method previously described [40], which measures the reduction in nitrobluetetrazolium (NBT) by superoxide ions. Human CuZn-SOD. Substrates used in these assays were obtained from Sigma Chemical Co. (St. Louis, MO, USA). Resulting data were analysed using the Newman−Keuls test.

## 5. Conclusions

In conclusion, we identified compounds **3**, **5**, **6**, **7**, **8** and **10** as potential fast killing drugs to fight against leishmaniasis. These tetradentate compounds exhibited a remarkable antileishmania effect against the two morphological forms of the parasite, even better than Glu, with a larger spectrum of action and lower toxicity.

Our studies suggest the possible mechanism of action of some of them: **3** and **5** seem to act at the glucose metabolism level of *L. infantum*; **6** and **7** involve the inhibition of the parasite SOD and the alteration in the glucose metabolism of *L. infantum* and *L. donovani*, respectively; **8** affects the glucose metabolism of *L. braziliensis*; and **10** damages the cellular ultrastructure on the three *Leishmania* spp.; without forgetting the powerful oxidizing of these compounds.

Based on their biological properties (leishmanicidal activity), **5** was the compound that demonstrated the best activity/selectivity profile against the two morphological forms of the three Leishmania spp. In general, they are promising molecules for the development of new leishmanicidal agents that could be implemented into a step further within the preclinical phase. Moreover, due to their mechanisms of action seems to be different, it merits mention that combined therapies (also using Glu) should be considered for obtaining improved efficacy.

Therefore, we present drugs for the development of an easy-to-synthesise, water-soluble and water-stable antileishmanial agents as a promising therapeutic alternative to current treatments.

## Figures and Tables

**Table 1:** In vitro activity, toxicity (**A**) and selectivity index (**B**) found for the polyaminic compound complexes on extracellular and intracellular forms of *Leishmania* spp.

Compounds	IC_50_ µM ^a^						Toxicity IC_50_ Macrophage (µM)
	*Leishmania infantum*		*Leishmania braziliensis*		*Leishmania donovani*		
	Promastigote Forms	Amastigote Forms	Promastigote Forms	Amastigote Forms	Promastigote Forms	Amastigote Forms	
Glucantime^®^	18.0 ± 3.1	24.2 ± 2.6	25.6 ± 1.6	30.4 ± 6.1	27.3 ± 4.3	33.3 ± 3.7	15.20 ± 1.3
1	18.5 ± 1.6	20.5 ± 1.8	22.3 ± 0.6	21.3 ± 0.9	28.6 ± 5.8	12.5 ± 2.7	37.9 ± 4.2
2	52.6 ± 3.1	57.5 ± 2.6	37.2 ± 1.9	12.5 ± 1.5	19.7 ± 1.7	16.3 ± 2.5	105.6 ± 8.8
3	8.5 ± 0.4	3.7 ± 1.6	9.3 ± 0.3	8.7 ± 0.6	28.6 ± 3.5	11.7 ± 1.3	98.5 ± 6.3
4	27.4 ± 0.7	24.7 ± 1.2	26.4 ± 0.7	19.4 ± 1.7	60.2 ± 7.1	29.9 ± 1.6	60.3 ± 3.3
5	1.3 ± 0.1	2.3 ± 0.8	3.5 ± 0.6	8.8 ± 1.3	6.8 ± 0.2	10.7 ± 0.8	97.2 ± 3.8
6	7.3 ± 0.1	10.2 ± 1.5	10.1 ± 3.1	11.7 ± 1.1	4.0 ± 0.1	1.3 ± 0.4	137.6 ± 7.4
7	12.3 ± 2.2	9.0 ± 0.4	11.7 ± 0.8	8.5 ± 0.3	8.1 ± 3.9	1.1 ± 0.2	172.1 ± 11.3
8	83.0 ± 9.9	22.5 ± 3.1	73.8 ± 3.6	21.7 ± 1.4	31.7 ± 2.4	1.9 ± 0.3	268.3 ± 16.2
9	71.7 ± 10.0	33.4 ± 3.2	53.2 ± 3.6	30.7 ± 2.5	29.5 ± 3.6	14.5 ± 2.7	100.5 ± 4.8
10	2.6 ± 0.7	10.4 ± 0.1	5.5 ± 0.8	11.9 ± 0.8	18.6 ± 0.9	11.6 ± 3.3	126.1 ± 7.1
11	87.5 ± 5.1	34.6 ± 2.5	60.6 ± 3.8	30.4 ± 1.3	29.5 ± 4.1	19.8 ± 2.6	75.1 ± 4.7
**SI ^b^**							
	***Leishmania infantum***		***Leishmania braziliensis***		***Leishmania donovani***		
	**Promastigote Forms**	**Amastigote Forms**	**Promastigote Forms**	**Amastigote Forms**	**Promastigote Forms**	**Amastigote Forms**	
Glucantime^®^	0.8	0.6	0.6	0.5	0.6	0.5	
1	2.1 (3)	1.8 (3)	1.7 (3)	1.8 (4)	1.3 (2)	3.0 (6)	
2	2.0 (2)	1.8 (3)	2.8 (5)	8.4 (17)	5.4 (9)	6.5 (13)	
3	11.6 (14)	26.6 (44)	10.6 (18)	11.3 (23)	3.4 (6)	8.4 (17)	
4	2.2 (3)	2.4 (4)	2.3 (4)	3.1 (6)	1.0 (2)	2.0 (4)	
5	74.8 (93)	42.3 (70)	27.8 (46)	11.0 (22)	14.3 (24)	9.1 (18)	
6	18.8 (24)	13.5 (22)	13.6 (23)	11.8 (24)	34.4 (57)	105.8 (212)	
7	14.0 (17)	19.1 (32)	14.7 (25)	20.2 (40)	21.2 (35)	156.5 (313)	
8	3.2 (4)	11.9 (20)	3.6 (6)	12.4 (25)	8.5 (14)	141.2 (282)	
9	1.4 (2)	3.0 (5)	1.9 (3)	3.3 (7)	3.4 (6)	6.9 (14)	
10	48.5 (61)	12.1 (20)	22.9 (38)	10.6 (21)	6.8 (11)	10.9 (22)	
11	0.9 (1)	2.2 (4)	1.2 (2)	(5)	2.5 (4)	3.8 (8)	

Results are averages of four separate determinations. ^a^ IC_50_ = the concentration required to give 50% inhibition, calculated by non-linear regression analysis from the Kc values at concentrations employed (from 100 µM to 1 µM). Each drug concentration was tested in triplicate. Values are the means of four separate determinations ± standard deviation. ^b^ Selectivity index = IC_50_ Macrophage Cell/IC_50_ extracellular and intracellular form of parasite. In brackets: number of times that compound exceeds the reference drug SI, on extracellular and intracellular forms of *Leishmania* spp.

## Supplementary Materials

The following are available online. Figure S1: Crystal structure of [Fe^II^_2_(TACN_2_)(CH_3_CN)_6_](ClO_4_)_4_ (**2**). Perchlorate anions have been omitted for clarity. (Selected bond distances: av. Fe-NCCH_3_ = 1.946 ± 0.004 Å, av. Fe-N_TACN_ = 2.038 ± 0.014 Å. Figure S2: Additional ultrastructural images of *L. braziliensis* promastigotes. Figure S3: Additional ultrastructural images of *L. donovani* promastigotes. Figure S4: Additional ultrastructural images of *L. infantum* promastigotes. Figure S5: ^1^H-NMR (400 MHz, CD_3_CN) of **2**. Figure S6: HRMS of **2**.
